# Application of Wearable Sensors Technology for Lumbar Spine Kinematic Measurements during Daily Activities following Microdiscectomy Due to Severe Sciatica

**DOI:** 10.3390/biology11030398

**Published:** 2022-03-03

**Authors:** Athanasios Triantafyllou, Georgios Papagiannis, Sophia Stasi, Daphne Bakalidou, Maria Kyriakidou, George Papathanasiou, Elias C. Papadopoulos, Panayiotis J. Papagelopoulos, Panayiotis Koulouvaris

**Affiliations:** 1 Orthopaedic Research and Education Center “P.N.Soukakos”, Biomechanics and Gait Analysis Laboratory “Sylvia Ioannou”, “Attikon” University Hospital, 1st Department of Orthopaedic Surgery, Medical School, National and Kapodistrian University of Athens, 12462 Athens, Greece; grpapagiannis@yahoo.gr (G.P.); hpapado@yahoo.com (E.C.P.); pjporthopedic@gmail.com (P.J.P.); info@drkoulouvaris.gr (P.K.); 2 Laboratory of Neuromuscular and Cardiovascular Study of Motion, Physiotherapy Department, Faculty of Health and Care Sciences, University of West Attica, 12243 Egaleo, Greece; soniastasi1@gmail.com (S.S.); dafbak@otenet.gr (D.B.); papathanasiou.g@gmail.com (G.P.); 3 Physiotherapy Department, University of the Peloponnese, 23100 Sparta, Greece; mariakyriakidou15@gmail.com

**Keywords:** lumbar spine biomechanics, wearable sensors, IMU technology, lumbar microdiscectomy, spine biomechanics

## Abstract

**Simple Summary:**

The recurrence rate after lumbar spine disc surgeries is estimated to be 5–15%. Lumbar spine flexion of more than 10° is mentioned in the literature as the most harmful load to the operated disc level that could lead to recurrence during the first six postoperative weeks. The purpose of this study is to quantify flexions during daily living following such surgeries, for six weeks postoperatively, using wearable sensors technology. These data determine the patients’ kinematic pattern, reflecting a high-risk factor for pathology recurrence. The operated patients were measured to have 30% normal lumbar motion after the first postoperative week, while they were restored to almost 75% at the end of the sixth, respectively. Further in vitro studies should be carried out using these data to identify if such kinematic patterns could lead to pathology recurrence.

**Abstract:**

Background: The recurrence rate of lumbar spine microdiscectomies (rLSMs) is estimated to be 5–15%. Lumbar spine flexion (LSF) of more than 10° is mentioned as the most harmful load to the intervertebral disc that could lead to recurrence during the first six postoperative weeks. The purpose of this study is to quantify LSFs, following LSM, at the period of six weeks postoperatively. Methods: LSFs were recorded during the daily activities of 69 subjects for 24 h twice per week, using Inertial Measurement Units (IMU). Results: The mean number of more than 10 degrees of LSFs per hour were: 41.3/h during the 1st postoperative week (P.W.) (29.9% healthy subjects-H.S.), 2nd P.W. 60.1/h (43.5% H.S.), 3rd P.W. 74.2/h (53.7% H.S.), 4th P.W. 82.9/h (60% H.S.), 5th P.W. 97.3/h (70.4% H.S.) and 6th P.W. 105.5/h (76.4% H.S.). Conclusions: LSFs constitute important risk factors for rLDH. Our study records the lumbar spine kinematic pattern of such patients for the first time during their daily activities. Patients’ data report less sagittal plane movements than healthy subjects. In vitro studies should be carried out, replicating our results to identify if such a kinematic pattern could cause rLDH. Furthermore, IMU biofeedback capabilities could protect patients from such harmful movements.

## 1. Introduction

Lower back pain is a common cause of disability worldwide, affecting more than 500 million people at any given time. Conservative treatment approaches typically involve combining medication with physical rehabilitation. Unfortunately, this option, in many cases, may not provide satisfactory treatment for patients, so surgery is an absolute indicator and the treatment option of choice. It is a fact that as high as 90% of lumbar spine microdiscectomies (LSMs) achieve good results during the first year postoperatively [1,2].

However, the reappearance of intervertebral disc herniation at the same level (recurrence lumbar disc herniation-rLDH) is among the most common causes of unsuccessful outcomes after lumbar microdissection surgery. The literature reports a recurrence rate of 5–15% [3,4,5,6,7]. The overall incidence of unsatisfactory results after primary lumbar microdissection is 5% to 20%, making recurrent hernia a significant cause of pain, disability, and in many cases, surgical repetition [8,9,10].

As a first step in reducing the recurrence rate, many studies have been conducted to identify the factors that may increase the risk. The literature reports age, gender, type of lumbar disc herniation, number of fragments removed, smoking, alcohol consumption, range of daily activities postoperative, and biomechanical factors [11]. There is essential evidence that postoperative lumbar spine flexion is an important risk factor for disc herniation, particularly when it exceeds 10° of sagittal plane range of motion. [11,12]. Patients with a sagittal plane range of motion (ROM) higher than 10° suffered from a recurrence rate of 26.5%, while those with less than 10° presented a rate of 4.1% [11]. 

Nowadays, kinematics of the spine is quantified mainly using optoelectronic cameras, fluoroscopic methods, video motion analysis, RGB cameras, and RGB-D sensors. [13,14,15]. The main disadvantage of such methods is that all data are collected in a laboratory environment, not reflecting the actual biomechanical behavior. As a result, much information is missing regarding the lumbar spine kinematic pattern during daily life where the patients are at risk of recurrence. 

Facing the absence of such essential data, for a detailed biomechanical understanding of rLDH, researchers suggest using the latest wearable technological innovations to provide closer, better, and more precise monitoring of patients undergoing such surgery.

The introduction of miniaturized sensors allows measuring lumbar spine kinematic patterns during daily activities by monitoring and recording patients’ movements for a prolonged time. Such measurements are critical for identifying and quantifying the possible effects of biomechanical factors on rLDH.

Moreover, existing wearable sensors solutions integrate biofeedback functions apart from measuring kinematics. An example is a vibrating ring in MetaMotionR+/Mbientlab sensors that could alert patients by applying vibration when they exceed ROMs, which can potentially be harmful to rLDH; as a result, this can protect them from risky movements [16].

Despite being a significant risk factor of rLDH, lumbar spine kinematics have not yet been measured in patients’ daily activities postoperatively. Therefore, the current study’s purpose is to quantify lumbar spine flexions, following microdiscectomy (microsurgical discectomy), during the everyday life of the subjects and observe the actual kinematic progress they present during the high-risk postoperative period by comparing these data to the measurements of normal subjects.

Such data combined with in vitro confirmation of dissected disc kinematics would allow the development of a strategy to control this risk by implementing the enhanced capabilities of wearable sensors technology. 

## 2. Materials and Methods

The current is an observational study aiming to quantify lumbar spine kinematics during daily activities in patients subjected to microdiscectomy at the level of the fifth Lumbar–first Sacral vertebrae (L5-S1) until the sixth postoperative week.

### 2.1. Subjects

Sixty-nine patients (43 male and 26 female) (Group B) that were subjected to lumbar microdiscectomy (Level–Lumbar 5th vertebrae to 1st sacral) because of sciatic pain and score 3+ (±1) of manual muscle test according to Medical Research Council scale (MRC) [17] included in the research. The mean age was 52.3 ± 13.2 years, and the mean body mass index (BMI) was 23.2 ± 2.6. 

Normal kinematic behavior during daily activities was measured at 43 healthy subjects (Group A) who presented no symptoms of low back pain or neurological signs and showed similar characteristics in terms of age and BMI to the operated subjects (Table 1).

### 2.2. Clinical Examination following Microdiscectomy

Evaluating an intervention’s effectiveness and a patient’s condition before and after treatment is quantified using various scales that measure physical disability. The most frequently used scale to assess the functional status of patients with low back pain is the JOA score, developed by the Japanese Orthopaedic Association in 1975 (Table 2) [18]. Our research evaluated all patients with a JOA score preoperatively and at the end of the 6th postoperative week.

### 2.3. Instrumentation and Procedure

Ranges of motion (ROMs) were recorded during the daily activities of subjects for 24 h twice per week from the first till the sixth postoperative week. Two MetaMotionR+, Inertial Measurement Units (IMUs) (CE-approved device for ROM measurements) were used to measure lumbar spine kinematics. MetaMotionR+ (MMR) is suitable for logging and streaming sensor data. Record raw sensor data were applied via Bluetooth at up to 400 Hz and stream raw sensor data at up to 100 Hz. Data were downloaded and accessed as a CSV file on the researcher’s computer.

The sensor fusion combines the measurements from a 3-axis gyroscope, a 3-axis geomagnetic sensor, and a 3-axis accelerometer to provide a robust absolute orientation vector in the form of Quaternion or Euler angles. In addition, algorithms intelligently fuse the raw sensor data to improve the output of each sensor. This procedure includes algorithms for offset calibration of each sensor, monitoring the calibration status, and Kalman filter fusion to provide distortion-free and refined orientation vectors.

MMR+ is a wearable device widely used for capturing continuous activity data motion and gesture tracking and includes the following sensors and technical characteristics:

BMI160 6-axis Accelerometer + Gyroscope
BMP280 Temperature
BMP280 Barometer/Pressure/Altimeter
LTR-329ALS Luminosity/Ambient Light
BMM150 3-axis Magnetometer
BOSCH 9-axis Sensor Fusion
8 MB Memory
Lithium-ion rechargeable battery
Vibrating Coin motor
Bluetooth Low Energy, CPU, button, LED, and GPIOs

A Bluetooth radio attached to each IMU wirelessly transmits data to a patients’ smartphone. The IMUs and Bluetooth radios are powered with 3.6 Volt batteries.

The whole procedure of data collection consists of six steps. 

#### 2.3.1. 1st Step—Calibration

The sensor frame and the body axis frame correlation must be identified to perform human motion analysis with inertial sensor technology [18]. Before powering and wearing the sensors, the IMUs were calibrated on a flat surface parallel to the ground to ensure that both sensors had the same zero reference coordinator. Therefore, the assumption that the 5th lumbar vertebrae and the sacral segments are in the same plane was considered correct. This allows for recording sensor data while the lumbar segment is aligned with one of the defined global frame axes. Following a solid calibration methodology is essential to collect reliable data for motion assessment. The calibration procedure took place using the Metawear, mbientlab INC application (software version: 2.0.1.) running on a smartphone.

#### 2.3.2. 2nd Step—Sensors Positioning 

**Figure 1:**
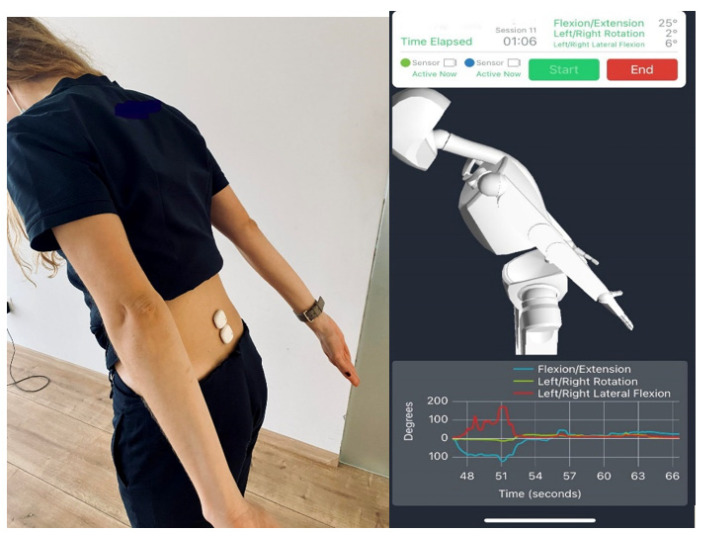
Wearable sensors application at the lumbar spine. Software reproduction–kinematic data recording.

The sensors were mounted as stable as possible to ensure the relative position and orientation between the sensor frame and axis of body movement were correctly determined [19,20]. The first IMU was placed just above the 1st sacral vertebra, and the second at the spinous process of the 5th lumbar vertebra. Both IMUs were attached to the skin using double-sided hypoallergic tape (Figure 1)

#### 2.3.3. 3rd Step—Bluetooth Protocol and Data Transmission

These IMU sensors communicate with a smartphone through Bluetooth protocol. Metawear, mbientlab INC application (software version: 2.0.1.) was installed to collect and store data in the smartphone. The application communicates and transmits recorded data via Bluetooth with a PC for further analysis after each session. Data streams in the local sensor coordinate system included in the analyses were: three-dimensional (3D) acceleration before and after filtering, magnetic sensing and orientation (roll, pitch, and yaw), and 3D angular velocity.

#### 2.3.4. 4th Step—Control of Test Measurements Trial Acquisition 

**Figure 2:**
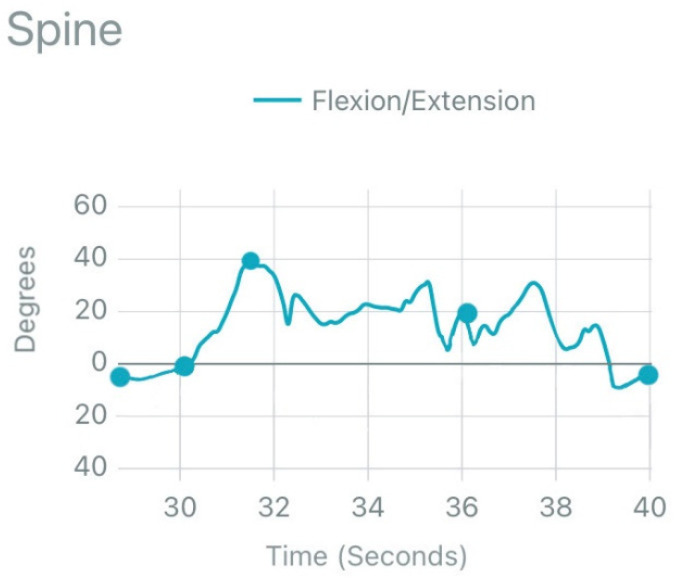
Biomechanical behavior of lumbar spine in the sagittal plane during stand up followed by ten steps walking in the calibration procedure.

To ensure the reproducibility of the procedure and that both wearable sensors functioned as intended and recorded a range of motion data, a stand and walk test was performed before collecting daily activities data. These data were saved on a CSV file and shared directly in the local drive -PC (one for each sensor). All subjects were prepared and given instructions about the procedure above. Next, they stood up from the chair and performed ten steps at a comfortable gait velocity. This trial was repeated three times to collect representative motion data. (Figure 2).

#### 2.3.5. 5th Step—Initial Data Interpretation 

Even if the accuracy of the used sensor is good and it uses root mean square error (RMS), a virtual human model allows the user to see if motion representation and data collection are being implemented correctly. When the sit and walk trial was completed, measured data were examined for unmeaningful results while the participants were still present. If errors or incorrect movements were found due to magnetic field interference, subjects did not leave the laboratory environment, and the calibration was checked to eliminate the disturbance.

#### 2.3.6. 6th Step—Daily Activities Acquisition

Upon satisfactory test trial, the patients were instructed to move at pain-free levels around the surgery area during daily life activities. In addition, they were asked to carry their smartphone close to their body during the “measurement day” to avoid Bluetooth signal connectivity issues during data transmission from the sensor to their smartphones. The following day (24 h of recording time), the patients visited the laboratory to disconnect the attached IMUs and transfer the measured data from the patients’ smartphones to the scientist’s computer. The whole procedure from the 1st to 6th step was performed and supervised by the same biomechanist.

### 2.4. Data Collection–Data Analysis

Two 9-axis IMU sensors were utilized to assess the number of lumbar spine flexions above 10 degrees. The sensors, placed on the patient’s spine can wirelessly transmit, via BLE, a three-axis tensor of the gyroscope component, containing the angle speed (deg/s) in axes x, y, and z. Similarly, a three-axis tensor of the accelerometer component, containing the acceleration (m/s^2^) in axes x, y, z, and a three-axis tensor of the magnetometer component, containing the magnetic field measurements in the same coordinate system.

Body segment orientation and corresponding Euler angles can be estimated accurately by fusing signals from gyroscopes, accelerometers, and magnetometers, which minimizes possible error sources [8]. 

MMR+ NDoF fusion algorithm performed automatic background calibration of the sensor. This is a 9 degrees of freedom mode, where the fused absolute orientation data is obtained from the accelerometer, gyroscope, and magnetometer. The advantage of combining all three sensors is a fast calculation, resulting in high output data rate, and increased robustness from magnetic field distortions. The fast magnetometer calibration option was turned ON in this mode, resulting in the rapid magnetometer calibration and increased output data accuracy. NDoF mode used the following frequencies for each component:


**Mode**

**Accelerometer**

**Gyroscope**

**Magnetometer**
NDoF100 Hz100 Hz25 Hz

The fusion algorithm uses the sensor’s accelerometer data to compensate for the gyroscope noise and signal drift over time. When the body segment of interest moves, the fusion instantaneously ignores the accelerometer data and relies on the gyroscope for pitch and roll. If the accelerometer data cannot be used for an extended period (due to constant movement or vibration), then this may cause the pitch and roll values to drift. Similarly, if the distortion affects the magnetometer data, the algorithm will automatically ignore it.

Let $\hat{x}$ = [a_x,a_y,a_z,g_x,g_y,g_x,m_x,m_y,m_z] be a single measurement of the IMU sensor. The first step is to estimate the Euler angles by utilizing the $\hat{x}$ measurements. Accelerometer, gyroscope, and magnetometer data are used to calculate the pointing vector of the L5 and the S1. Thus, the data a x, g x, and m x are translated to Euler angles of Pitch plane as they correspond to lumbar spine flexion angles [21].

For this, the methodology proposed by Qifan Zhou et al. 2021 [22] was applied, enabling a Kalman filter to estimate the absolute orientation of the sensing device. The Kalman filter is a closed-loop system used to model nonlinear systems, which deal with cases governed by nonlinear differential equations. It consists of four steps: Prediction, Kalman gain, Update, and Quaternion normalization. The IMU sensor input is provided during the update step, and after each of the four steps is executed, the output is provided as an orientation estimation [21,23]. 

Finally, standard Euler angles were [23] used to define the lumbar spine flexion angle in relation to the sacral coordinate system using sampling rates of the IMUs system at 5 Hz. 

Based on the Euler angles estimated by the approach mentioned above, a model for assessing the 10 degrees flexions is employed. First, a zeroing pre-processing step is applied, assuming that the most frequent angle in the pitch plane refers to the body’s upright position. Based on this outcome, after relatively translating all the collected values, the number of instances a difference of more than 10 degrees occurs is calculated. Finally, a sliding window post-processing algorithm parses the output results and eliminates measurements closer than 5 s.

## 3. Results

### 3.1. Clinical Examination following Microdiscectomy:

#### The Japanese Orthopaedic Association (JOA) Score

**Figure 3:**
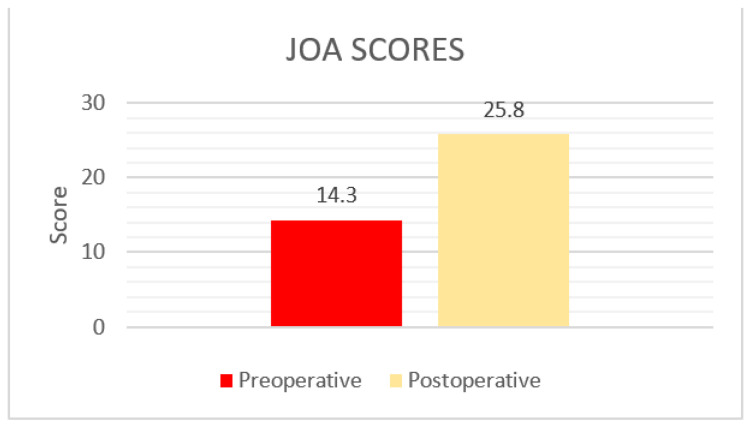
The Japanese Orthopaedic Association (JOA) scores pre and postoperatively.

The JOA score evaluated the patients’ leg pain, low back pain, and daily activities. In this system, the maximum score is 29 points, and higher JOA scores refer to better results regarding the parameters evaluated [24]. The average preoperative JOA score was 14.3 (Standard Deviation (SD) = 2.8) (Group B). Six weeks postoperatively JOA score was evaluated at 25.8 (SD = 4.2) for the same group (Figure 3).

### 3.2. Wearable Sensors–IMU Data

**Figure 4:**
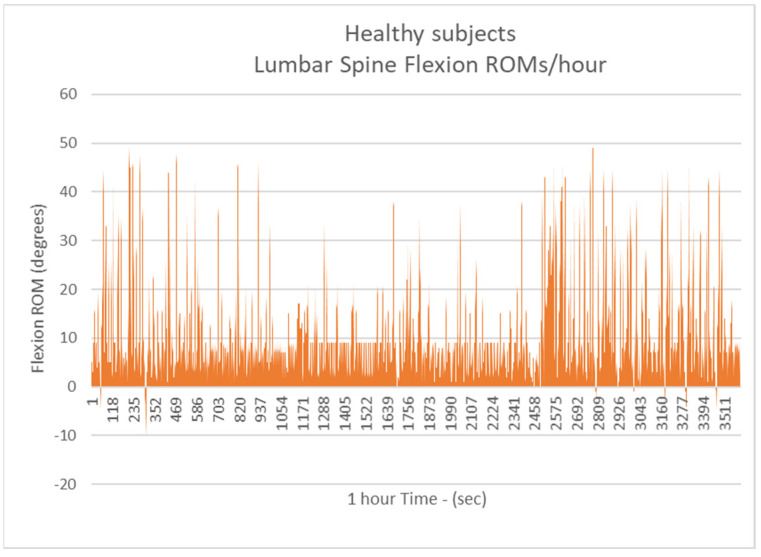
The number of more than 10 degrees of Lumbar Spine Flexions (LSFs) per hour for healthy subjects.

The mean number of more than 10 degrees of Lumbar Spine Flexions (LSFs) per hour was 138.16 for healthy subjects (H.S.) (Figure 4) (Group A).

**Figure 5:**
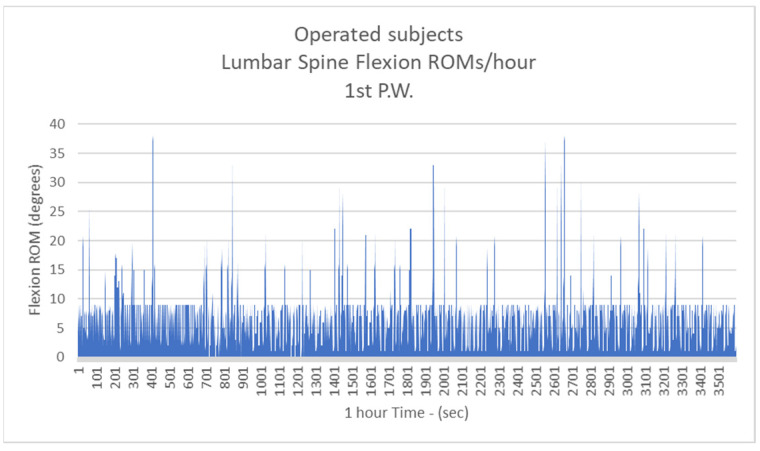
The number of more than 10 degrees of Lumbar Spine Flexions (LSFs) per hour for operated subjects at the 1st postoperative week.

**Figure 6:**
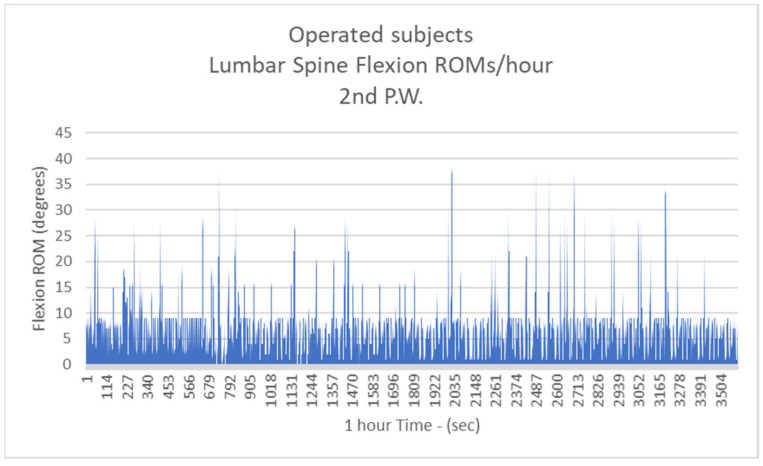
The number of more than 10 degrees of Lumbar Spine Flexions (LSFs) per hour for operated subjects at the 2nd postoperative week.

**Figure 7:**
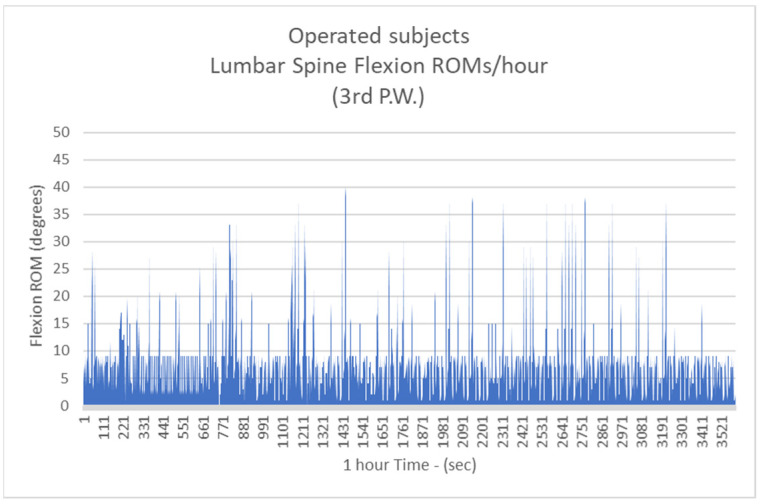
The number of more than 10 degrees of Lumbar Spine Flexions (LSFs) per hour for operated subjects at the 3rd postoperative week.

**Figure 8:**
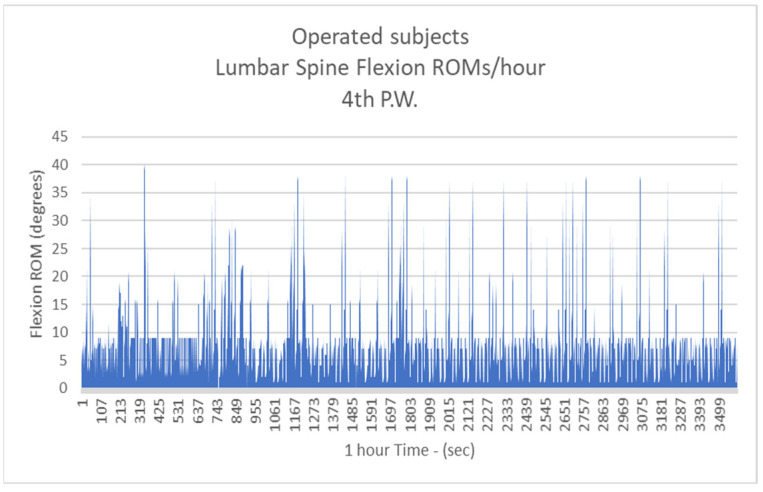
The number of more than 10 degrees of Lumbar Spine Flexions (LSFs) per hour for operated subjects at the 4th postoperative week.

**Figure 9:**
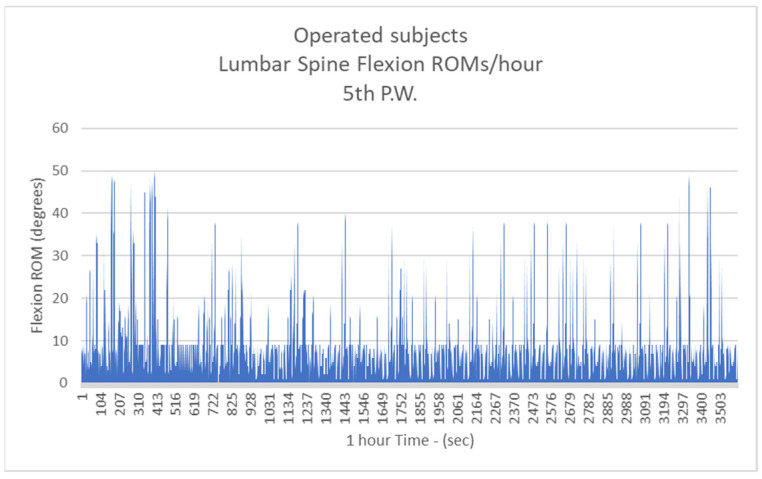
The number of more than 10 degrees of Lumbar Spine Flexions (LSFs) per hour for operated subjects at the 5th postoperative week.

**Figure 10:**
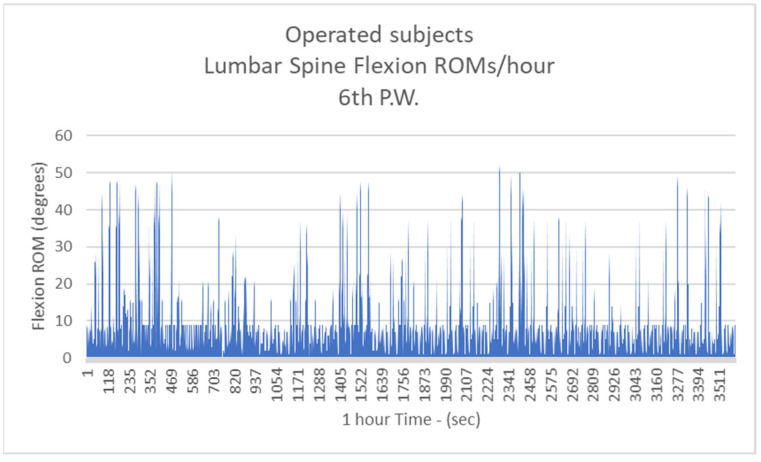
The number of more than 10 degrees of Lumbar Spine Flexions (LSFs) per hour for operated subjects at the 6th postoperative week.

Respectively, the mean number of more than 10 degrees of LSFs per hour for Group B were:

- 41.3/h during the 1st postoperative week (P.W.) (29.9% healthy subjects-H.S.) (Figure 5);
- 2nd P.W. 60.1/h (43.5% H.S.) (Figure 6);
- 3rd P.W. 74.2/h (53.7% H.S.) (Figure 7);
- 4th P.W. 82.9/h (60% H.S.) (Figure 8);
- 5th P.W. 97.3/h (70.4% H.S.) (Figure 9);
- 6th P.W. 105.5/h (76.4% H.S.) (Figure 10).

**Figure 11:**
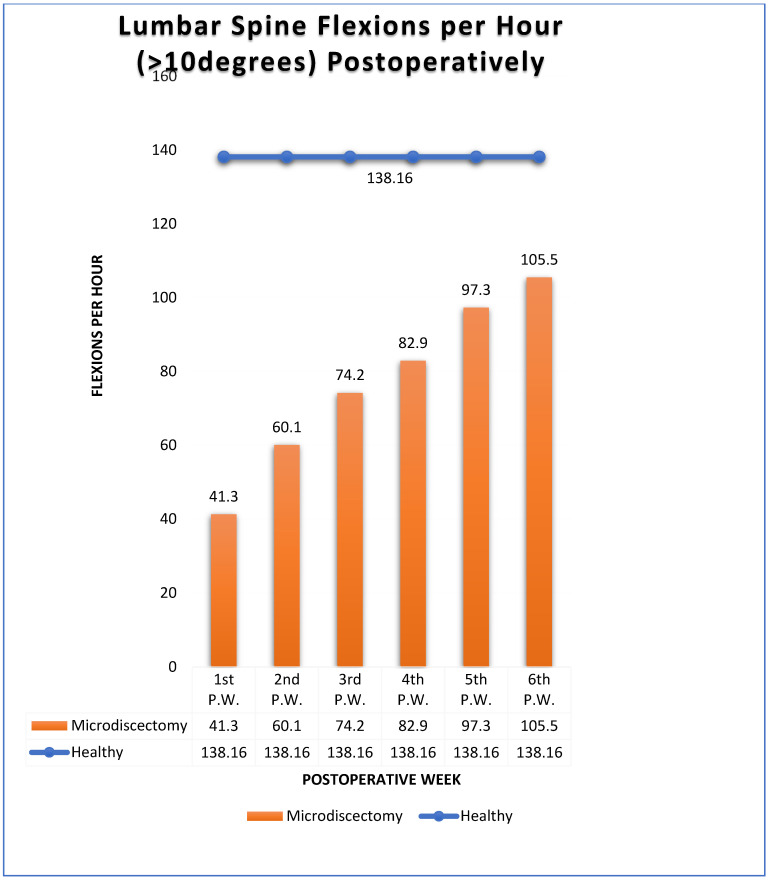
Results presenting the number of lumbar spine flexions (more than 10 degrees) for healthy subjects (blue line) and operated patients (orange bars).

Figure 11 shows cumulatively the number of more than 10 degrees of Lumbar Spine Flexions (LSFs) per hour for operated and healthy subjects.

**Figure 12:**
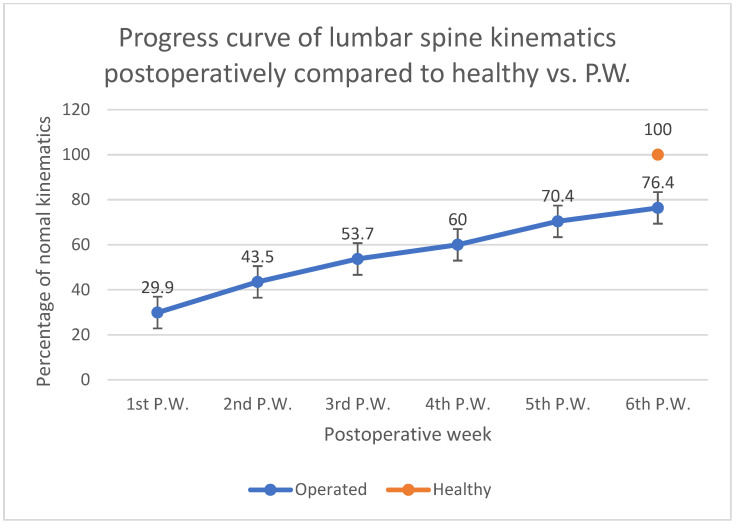
Percentage evolution of lumbar spine kinematics postoperatively compared to healthy, in relation to postoperative week.

The results show that the number of lumbar spine flexions more than 10 degrees present a linear increase from the 1st to the 6th postoperative week. Nevertheless, at the end of the 6th postoperative week, the patients restored close to 75% of the normal spine kinematic behavior (Figure 12). 

## 4. Discussion

Quantitative data on vertebral motion is critical to understanding spinal pathology, improving current surgical treatments of spinal diseases, and controlling rLDH [25]. The essential biomechanical factor for rLDH mentioned in the literature is sagittal plane ROMs [11,26]. More than 10° of lumbar spine flexion postoperatively was responsible for a recurrence rate of 26.5%. Patients with less than 10° had a rate of 4.1% [11]. Even if the rate of sagittal ROMs has been identified, the actual number (frequency) that occurs during daily activities postoperatively has not been measured. In our study, lumbar spine kinematics following microdiscectomy was recorded during everyday activities for 24 h till the sixth postoperative week. Such kinematics quantification due to exposure to repetitive loads was recorded for the first time to our knowledge. The subjects were limited to 44% of the normal (healthy subjects) flexion kinematics (more than 10 degrees) at the 1st postoperative week and improved to 76.9% at the sixth postoperative week.

Until the writing of this manuscript, only one study investigated the monitoring of patients following lumbar spine surgery, to our knowledge. Mijailovic and his colleagues in 2012 [27] proposed a method to calculate ROM values of lumbar spine motions of patients who had undergone spine surgery by using wireless three-dimensional acceleration measurements. A possible weakness of such a study concerns the error value and measurement uncertainty. The authors identified the potential sources of errors as the measuring sensor’s final resolution and the error resulting from signal filtering averaging. Considering the devices’ characteristics and the mean average deviation for all performed measurements, they found that the total error was limited to the range of 1.5°. This study indicates how sensors technology could accurately monitor kinematic patterns following lumbar disc microdiscectomy. 

As a first step in reducing the recurrence rate, many studies have been conducted to identify the factors that may increase the risk. The main factors mentioned in the literature are age, gender, type of lumbar disc herniation, number of fragments removed, smoking, alcohol consumption, range of daily activities postoperative, and biomechanical factors [28]. A significant biomechanical factor that can cause a disc herniation to recur after discectomy is when the fibrous ring at the point of hernia removal has not healed completely, thereby allowing this attenuated load-bearing point to continue to be mechanically exposed. Biomechanical risk factors for recurrent intervertebral disc herniation reported in the literature include reduced resistance to fibrous ring loads and exposure to repetitive loads caused by weightlifting or vibration [29,30,31,32,33,34]. In addition, the literature review provides essential evidence that lumbar spine flexion represents a significant risk factor for disc herniation [11,12]. 

Wade et al. conducted a study of the microstructural investigation of the disruption caused by bending lumbar disc compression. The study’s conclusions indicate that herniation can be induced when compressed loads are exerted on the bent disk. The injury begins with ruptures in the middle and outer layers of the fibrous rings as a possible consequence of the aforementioned change [35]. Other studies report that patients with intervertebral disc herniation exhibited greater compression forces across all lumbar intervertebral discs during trunk flexion, which increased with higher flexion angles [36]. Although these results correlate with the previous study demonstrating the harmful effect of kinematics (lumbar spine flexion- bending) on these patients, they did not quantify the actual number of flexions needed for such a result. Our study measured these risk factors in real-life activities in an effort to provide the data missing to predict rLDH. 

In a recent study, Costi and colleagues [37] found that when repeated loads are applied to degenerated intervertebral discs in combination with compression and bending or bending and axial rotation, which is the most typical way we lift weights, maximum shear stress is developed. That can subsequently cause rupture of the fibrous rings and detachment of the gel from the kinetic plate. Therefore, researchers suggest the above may indicate possible intervertebral disc herniation. These results agree with those of Eun Sang Soo et al., who identified intervertebral instability and a greater than 10-degree change of flexion angle as a possible factor for rLDH [38]. Cadaveric studies have simply measured lumbar segment motion by applying flexion–extension, bending, and axial rotational torques, with or without a compressive load [25,39] without involving the actual daily life kinematic pattern. On the other hand, in vivo motion of the lumbar segments has often been evaluated using imaging techniques to capture the lumbar vertebrae positions in different static postures [40] or by analyzing motion with the use of optoelectronic cameras of fluoroscopy in a laboratory environment. While these studies have significantly improved our knowledge of lumbar motion, in vivo kinematics of lumbar vertebrae during daily activities remain largely unexplored using 3D measurement techniques. Therefore, in everyday life, accurate dynamic motion characteristics of L4–5 and L5–S1 are still not clearly described in the literature [25]. Recently, a 3D fluoroscopic imaging technique has been intensively applied to investigate 6 degrees-of-freedom (6DOF) lumbar kinematics during various weight-bearing static end-range postures and dynamic axial rotation once more in a laboratory environment [40]. The detailed recording of this kinematic pattern should be tested in vitro, using cadaveric biomechanical testings, to identify the actual postoperative week that rLDH potentially occurs [41]. Our study measured the frequency of such kinematics during daily activities of patients subjected to lumbar microdiscectomy. We believe that if we recognize that time frame, we could control such kinematic risk factors by taking advantage of biofeedback capabilities of wearable sensor devices to alert and protect patients from harmful ROMs. An example is a vibrating ring in MetaMotionR+/Mbientlab sensors (the devices we used in our study) that could alert patients by applying vibration when they exceed ROMs, potentially harmful to rLDH; as a result, protect them from risky movements.

Surgical interventions following a degenerative disease alter vertebral load properties and lumbar spine biomechanics, increasing the risk of recurrent disc herniation. Kim et al., in their study, examined biomechanical factors that potentially contribute to rLDH using preoperative imaging [11]. They concluded that patients with sagittal plane ROM more than 10° had a recurrence rate of 26.5%, while those with less than 10° presented a rate of 4.1%. Kyoung-Tae Kim et al. [36] report that sagittal lumbar spine ROM correlated significantly with a high incidence of rLDH. On the other hand, Zhonghai Li et al. [26] mention that research about sagittal axle range of motion on rLDH (sROM) rarely appears in the literature. Our data measured this parameter in daily activities for the first time, thus providing the necessary information to biomechanists to identify whether and which frequency of more than 10 degrees of sROM could constitute a biomechanical risk factor for rLDH. 

Advances in the development of tiny sensors have created new possibilities for long-term recording and quantification of human body kinematics, including assessments in everyday life environments where motor disorders can be spontaneously developed and reproduced by the subjects. A typical example is the study of back pain using sensors both in the workplace [42,43,44,45,46,47,48,49,50] and in the daily lives of users [51,52,53], which is the pathology responsible for the most considerable loss of labor hours in the western world [54,55]. Evaluation of biomechanical factors can positively impact health care by better understanding the underlying mechanical factors that could cause back pain or rLDH. 

Portable and wearable sensors, especially inertial sensors, have gained immense popularity in biomechanical studies of motion in a short time [56]. Their properties such as lightweight, small size, low cost, energy efficiency, and portability make them suitable for a variety of applications, from the simple recording of daily activities [50,57] to the most complex kinematic physical activity in a laboratory environment [58,59], but mainly in the daily lives of users.

Continuous monitoring of spine motion provides the opportunity for objective and quantitative analysis of kinematics, which offers insight into how spinal movements affect mechanical changes and, hence, disc herniation development. [16]. Our study provided such valuable data for patients subjected to lumbar spine microdiscectomy for the first time. This can evaluate the relationship between the disc re-herniation trigger mechanism and daily activities. Such measurements in patients’ daily lives could help promote the prevention by implementing wearable sensors’ biofeedback capabilities and reducing exposure to mechanical risk factors. They could also support user monitoring, thereby facilitating and encouraging patient self-management.

## 5. Conclusions

Our study aimed to measure the number of lumbar spine flexion instants for six weeks postoperatively, a risk factor (lumbar flexion) for rLDH after microdiscectomy in the lumbar spine. These are the kinematic parameters revealed by this literature review that provide the most harmful load to the intervertebral disc and could lead to a recurrence of the pathology during the six postoperative weeks, referred to as a “collapse” point, due to the load mentioned above. Therefore, following our results of identifying patients’ kinematic pattern during everyday activities and quantifying the overall number of flexions, in vitro studies should be conducted to verify if these data results can cause reherniation of microdiscectomied lumbar discs.

Moreover, wearable sensors could be used, apart from monitoring kinematics, as biofeedback solutions, by applying vibration when patients exceed ROMs, which constitute a biomechanical risk factor for rLDH and, as a result, protect them from risky movements.

## Figures and Tables

**Table 1:** Demographic characteristics of healthy and operated subjects.

Item		Average (±Standard Deviation)	
		Healthy	Operated
Total number		43	69
Gender	Male	24	43
	Female	19	26
MRC test		5	3 (±1)
Age		49.1 ± 11.7	52.3 ± 13.2
BMI		22 ± 2.1	23.2 ± 2.6
Level			L5-S1
JOA Score	Preoperative		14.3 ± 2.8
	Postoperative		25.8 ± 4.2

**Table 2:** The Japanese Orthopaedic Association Score.

Item Evaluated	Score Range
Subjective Symptoms (9 points)	
➢ Low back pain ➢ Leg pain and/or tingling sensation ➢ Walking ability	➢ 3,2,1,0 ➢ 3,2,1,0 ➢ 3,2,1,0
Objective symptoms (6 points)	
➢ Straight leg raising test ➢ Sensory disturbance in the lower extremities ➢ Muscle weakness in the lower extremities	➢ 3,2,1,0 ➢ 3,2,1,0 ➢ 3,2,1,0
Restriction of daily activities (14 points)	
➢ Turning over while lying ➢ Keeping standing ➢ Face-washing ➢ Kneeling position ➢ Lifting or holding heavy object ➢ Walking	➢ 2,1,0 ➢ 2,1,0 ➢ 2,1,0 ➢ 2,1,0 ➢ 2,1,0 ➢ 2,1,0
Urinary bladder function	
	➢ 0, −3, −6
Total Score	➢ 29 to −6

## Data Availability

Data available on request due to restrictions of privacy.

## Abbreviations

ISOS	International Surgical Outcomes Study
LSMs	Lumbar Spine Microdiscectomies
rLDH	recurrence Lumbar Disc Herniation
ROM	Range of Motion
L5-S1	Fifth Lumbar–First Sacral vertebrae
MRC	Medical Research Council scale
BMI	Body Mass Index
JOA	Japanese Orthopaedic Association
IMU	Inertial Measurement Units
SD	Standard Deviation
LSFs	Lumbar Spine Flexions
N.S.	Normal Subjects
PW	Postoperative Week
